# Significance of ^18^F-FDG PET/CT in Characterization of Equivocal Lesions in High-Risk Testicular Carcinoma in Restaging Setting

**DOI:** 10.31557/APJCP.2020.21.2.511

**Published:** 2020

**Authors:** Rashid Rasheed, Fareeda Al-Kandari, Mohammad Ghanem, Fahad Marafi, Sharjeel Usmani

**Affiliations:** 1 *Department of Chemistry, Government College University (GCUF), Kotwali Road, Gurunanakpura, 38000, Faisalabad, Punjab, Pakistan, *; 2 *Department of Nuclear Medicine, Kuwait Cancer Control Center, Ministry of Health, *; 3 *Department of Nuclear Medicine, Faculty of Medicine, Kuwait University, *; 4 *Department of Nuclear Medicine, The Jaber Al-Ahmad Center for Molecular Imaging and Nuclear Medicine, Kuwait. *

**Keywords:** Testicular cancer, FDG in characterization of lesions, high-risk testicular carcinoma

## Abstract

**Objectives:**

The present study aims to evaluate the role of Positron emission tomography (PET) -computed tomography (CT) with ^18^F-fluorodeoxyglucose (^18^F-FDG) in the restaging of high-risk testicular cancer.

**Methods:**

Forty-five patients (mean age of 38.1±11.3 years and range 23-81 years) with testicular carcinoma, underwent ^18^F-FDG PET-CT during their clinical course were prospectively selected. PET positivity was defined as a site of abnormal ^18^F-FDG uptake in tissue histologically proven or clinically or radiographically suspected to represent tissue involvement. The sites of disease were characterized as either nodal or extranodal. All patients were followed-up for at least 12 months with a diagnostic and/or functional imaging modality.

**Results:**

Of the 45 patients 38 (84%) patient presented with seminoma and 7 (16%) were Non-seminomatous germ cell tumors. Analysis of secondary disease spectrum showed nodal involvement in 65%, osseous involvement in 23% and mixed visceral/soft tissue lesions in 12% of patients. Nineteen (42%) were negative for any metastatic disease. All negative patients remain disease free in the follow-up of one year. Out of the positive 26/45 patients, PET-CT showed progressive disease in 3/26, stable disease 1/26 and partial response in 2/26 and complete metabolic resolution in 20/26 patients. ^18^F-FDG PET-CT was able to characterize all patients leading to significant change of primary decision of wait and watch to go for treatment and vice versa.

**Conclusion:**

^18^F-FDG PET-CT scan is potentially an excellent tool for characterization of equivocal lesions on CT scan in the restaging settings and follow up of high-risk testicular cancer patients.

## Introduction

Positron emission tomography (PET)/computed tomography (CT) with ^18^F fluorodeoxyglucose (^18^F-FDG) has become pervasive as a tool for staging, restaging and follow-up of testicular cancer patients, especially in characterization of equivocal lesions on ultrasound (US), CT and MRI (Els et al., 2010). Testicular cancer represents between 1% and 1.5% of male cancers and 5% of urologic tumors, with 3 to 10 new cases occurring per 100,000 males per year (Albers et al., 2012). High risk factors include young age, prior family history, HIV, undescended testes, carcinoma In-situ and being white man. Over the last 30 years, the incidence of testicular cancer has increased (Manecksha et al., 2009). Bilateral presentation is low i.e.1% to 2% at diagnosis. There is a clear predominance (90%–95%) of germ cell tumors (GCT) despite of overall variation in presentation. Testicular cancers are classified as seminomas, accounting for approximately 40% of GCT or non-seminomatous GSTs (NSGCT), which account for approximately 60%. Pathology, staging, and prognostic stratification are main determinants of testicular GCTs for their clinical management (Horwich et al., 2006; Flechon et al., 2008). 

In a study by Jana and Blaufox (2006) she showed that main route of spread of testicular cancer is through lymphatic vessels to the retro peritoneum. Lungs are the main organs of hematogenous spread of testicular cancer. Testicular cancer normally presents as a painless, unilateral scrotal swelling. In approximately 20% of cases, the initial symptom is scrotal pain. 

Orchiectomy and pathologic examination of the testes are mandatory to confirm the diagnosis and to define the local spread. Serum tumor markers (alpha fetoprotein, human chorionic gonadotropin, and lactate dehydrogenase) are prognostic factors and contribute to diagnosis and staging.

Imaging plays an important role in the clinical management of testicular cancer. Initial imaging is performed using ultrasonography (US) to evaluate the size, location, solidness, bilateral synchronous tumors and calcifications in the tumor. Doppler flow helps to differentiate benign vs malignant mass based on blood flow images. US also helps in image guided biopsies and characterization of the equivocal liver lesions on CT (Grantham et al., 1985). Chest x ray is initially performed to rule out lung metastases as it can easily pick tumor >1cm.

MRI is less likely used in staging and restaging of the testicular cancer due to its high cost and less availability however, Janet et al., (2004) showed that it still carries same accuracy as of CT scan in detection of the metastatic lesions.

It is well documented that CT alone cannot differentiate necrosis / fibrosis from viable tumor in residual disease post chemotherapy especially in lesions <3cm whereas its accuracy is 100% for masses >3 cm and 95% for masses <3 cm (Zhao et al., 2014). Other pitfalls include Large gonadal veins, duplication of the inferior vena cava, left-sided inferior vena cava, retro-aortic and circumaortic renal vessels and left ascending lumbar communicating veins. Thoracic CT is most sensitive in localization of the lung lesions however it cannot differentiate viable from non-viable tumor lesions.

^18^F-FDG PET/CT has become one of the most important imaging modalities for patients with testicular cancer especially it serves as ubiquitous as a tool for staging, re-staging, characterization of lesions and follow-up of testicular cancer patients.

In this study, we focus on the role of ^18^F-FDG PET CT in patients with high risk of recurrence, who were post chemotherapy for last one year or in those patients where CT showed equivocal findings. 

## Materials and Methods

The current study was approved by hospital research and ethical committee and all patients gave informed consent. We prospectively selected the patients who underwent ^18^F-FDG PET-CT during their clinical follow up at our institution (Table 1).

 The following data were recorded: age, sex, histologic type, study indication, radiology results, sites of disease, and maximum standardized uptake value (SUV max). PET-CT positivity was defined as a site of abnormal FDG uptake in tissue histologically proven or clinically or radiographically suspected to represent tumor involvement. The cases were sorted according to the types of referral as shown in table 1. Our total population comprised of 45 patients with a mean age of 38.1±11.3 (23-81) years. All patients were followed-up for at least 12 months with a diagnostic and / or functional imaging modality. The patients referred for PET CT included those who underwent prior imaging using (CT, MRI, US) and had either equivocal lesions and or for characterization of a definite lesion seen on either imaging modality with non-diagnostic biochemical markers. Patients with no follow-up or in those where follow-up imaging was considered non-diagnostic were excluded. All patients were followed for one year. Follow up, clinical assessment, biochemical results (LDH), tumor markers (βHCG and/or AFP) and histopathological correlation (where ever available) were considered as gold standards for interpretation and characterization of lesions.

^18^ *F-FDG PET-CT PROTOCOL*

All patients underwent ^18^F-FDG PET-CT systems (Discovery 690 and 710, from GE Healthcare). Images were acquired after IV injection of 0.06 mCi/kg FDG and a 60- to 90-minute uptake period. Blood glucose level was less than 200 mg/dL before injection. PET emission images were obtained at 2-3 minutes per bed position according to patient BMI from the vertex to toes. PET, CT, and fusion images were reviewed by two board certified nuclear physicians on a workstation integrated with a PACS on Hermes Hybrid viewer version 2.2.

## Results

Demographic analysis /general characteristics of the patients are described in Table 1. Total 45 patients were included in the study for restaging of high-risk testicular cancer. Mean age of the cohort was 38.1±11.3 with a range of 23 to 81 years. Mean weight, fasting blood sugar and injected dose of population was 89.9±24.5Kg, 5.86±1.84 mmol/L and 5.63±1.96 mCi respectively. There were 22 Kuwaiti and 23 non-Kuwaiti patients with predominantly resented with seminoma 38/45 (84%) and Non-seminoma in 7/45 (16%) of total population. Analysis of secondary disease spectrum showed equivocal para-aortic lymphadenopathy 17/45 (38%), rising tumor markers with negative CT scans 06/45 (13%), characterization of pulmonary and hepatic lesions 13/45 (29%), indeterminate residual masses/surveillance in higher risk patients of patients 09/45 (20%).

Of 45 patients analyzed, in 19/45 ^18^F-FDG PET/CT were unremarkable. All negative patients remain disease free in follow-up of one year as ^18^F-FDG PET/CT has negative predicted value of 100%. However out of positive 26/45 patients, post treatment follow up ^18^F-PET CT showed progressive disease in (n=03/26), stable disease in (n=01/26) and partial response in (n=2/26) and complete metabolic response in 20/26 patients. None of the PET CT scans were categorized as non-conclusive. 

**Table 1:** Patient Characteristics

Parameters	Mean (SD) / Frequency (%)
Mean Age (range)	38.1±11.3(23-81) years
Weight	89.9±24.5
Fasting blood sugar (FBS)	5.86±1.84
Dose	
(MBq)	208.41±72.7
(mCi)	5.63±1.96
Nationality	
Kuwaiti	22 (49)
Non-Kuwaiti	23 (51)
Type of tumor	
Non-Seminoma (NSGCT)	7(16)
Seminoma	38(84)
Primary Site	
Right sided	17(38)
Left sided	28(62)
Types of referral of equivocal lesions	
Equivocal para-aortic lymphadenopathy	
Characterization of pulmonary and hepatic lesions	
Indeterminate residual masses/surveillance in higher risk patients	
Rising tumor markers with negative CT scans	

**Figure 1:**
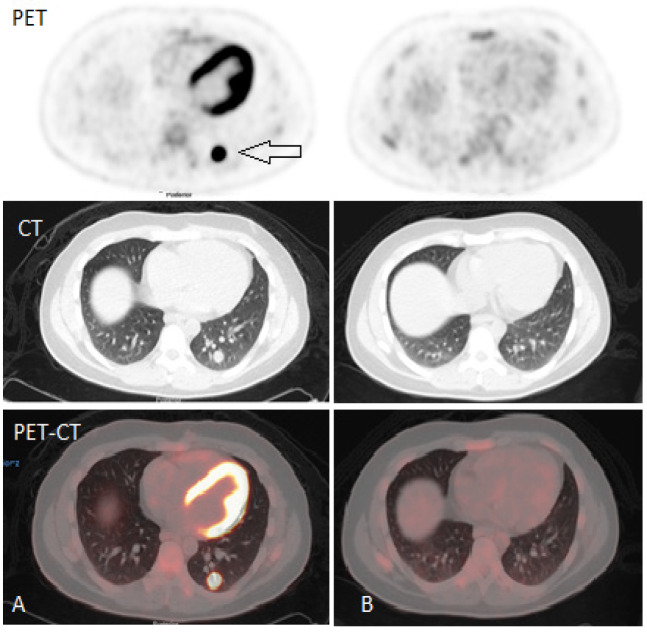
23-Years Old Male with Testicular Cancer. Right sided orchidectomy done one year back. CT scan showed solitary lung nodule on the left side. Further characterization was requested through FDG PET-CT scan (injected dose=4.7mCi) which showed hyper metabolic pulmonary nodule (A) suggestive of metastatic disease relapse. B) post therapy image show complete resolution

**Figure 2:**
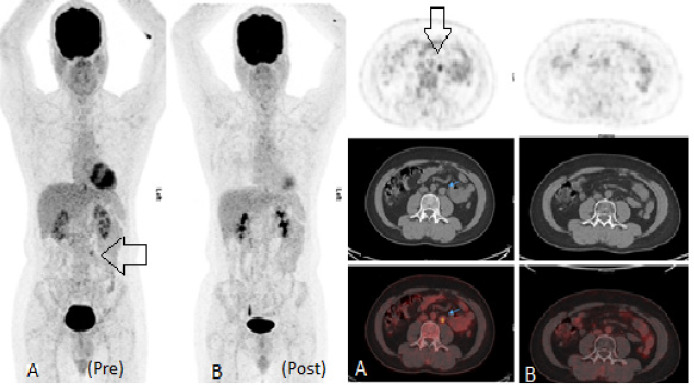
44-Years Man Underwent Orchidectomy for Seminoma of Left Testicle One Year Back. Follow up imaging showed solitary para-aortic lymph node which was difficult to characterize on CT scan as it was < 3cm, however FDG PET (A) confirmed its viability (arrows in Figure A) leading to start of treatment. (injected dose= 5.0 mCi) B) Post therapy PET CT showing complete resolution

**Figure 3:**
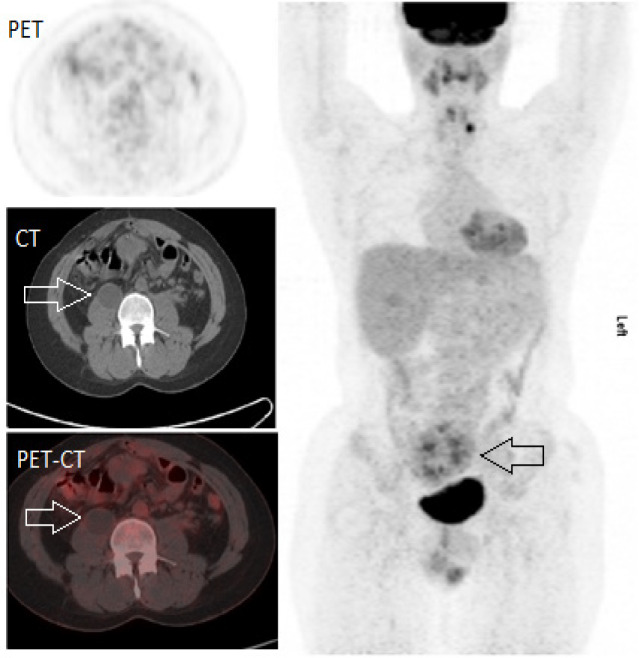
48-Year Old Man Underwent Follow up CT Scan Showing Cystic Lesion at the Right Psoas Muscle (white arrows). 18F-FDG PET CT showed negligible FDG uptake in the cystic lesion consistent with benign lesion

## Discussion

Testicular tumors show excellent cure rates after proper therapy. This is mainly owing to careful staging at the time of diagnosis, adequate early treatment based on chemotherapeutic combinations with or without radiotherapy and surgery, and very strict follow-up and salvage therapies. Tumor markers (βHCG and/or AFP) are elevated at relapse in about 2/3 of NSGCT and approximately 1/3 of seminomas. While LDH is vital in prognostication of metastatic disease and thus should be routinely included, however its use in detecting relapse is questionable (Ackers et al., 2006).

Tamer and Kassem (2016) showed that ^18^F-FDG PET/CT has a major role in preoperative staging of testicular tumors and defines the need of post-operative adjuvant therapy. However, Santis et al., (2004) showed that in restaging settings the sensitivity and specificity of ^18^F-FDG PET/CT in detecting residual/recurrent testicular tumor is 80% and 100% respectively when compared to CT alone which is 70% and 74% respectively. 

The fact that relapses are discernible, therefore serum tumor markers and/or radiological imaging confers a responsibility for their prompt detection. However, false-negative or false positive CT examinations can occur owing to the inability of this modality to detect foci of viable disease in normal-sized nodes or to differentiate benign from malignant enlargement (Thomas et al., 1981). For practical purposes then, a cutoff of 10 mm on CT is used, treating those measuring between 8 and 10 mm as suspicious/equivocal. These measurements are to be taken in the overall context of the patient (risk of disease, markers and tumor laterality) (Dalal et al., 2006). In NSGCT, usual imaging modalities are unable to differentiate in fibrosis/necrosis from viable metastases with further inability to characterize solitary lung nodule on follow up CT scan. ^18^F-FDG PET/CT has shown a vital role in characterization and differentiation of viable metastases/equivocal lesion from benign/fibrotic tissue residues. 

Of total 45 cases included in the study, 39/45 (87%) patients were referred for ^18^F-FDG PET/CT for characterization of the definite but equivocal lesions seen on CT. ^18^F-FDG PET/CT was targeted to characterize these lesions, hence changed the management from wait and watch to go for treatment. Furthermore, cases with rising tumor markers with negative CT scans (n=6) were also included in the study for establishing definitive diagnosis.

In a 23-years old male, CT alone was unable to characterize the viability of focal 10 mm nodule in the lower lobe of left lung as shown in Figure 1. Further characterization with ^18^F-FDG PET/CT was advised to exclude viability. The nodule showed good FDG uptake and appeared viable (1A), hence leading to the change in decision, as to start treatment rather than to put on follow up as per CT criteria of solitary lung nodule. These results are in accordance with the multicenter SEMPET trials where (Santis et al., 2004; Bachner et al., 2012 and Hinz et al., 2012) showed that ^18^F-FDG PET/CT can successfully differentiate post chemotherapy residual or recurrent equivocal lesions.

 Becherer et al., (2005) showed that restaging FDG PET/CT is better than CT alone in prediction of viable tumor after completion of chemotherapy as seen in a 52-year old man with para-aortic recurrence as seen in Figure 2A. Here ^18^F-FDG PET scan characterized this sub-centimetric lesion as viable and patient received treatment. Follow up post treatment PET scan showed complete metabolic resolution (Figure 2B). Our results are also in congruence to study published by Spermon et al., (2002) who showed that FDG PET-CT can better characterize the response than other modalities. 

Stomper et al., (1991) showed that FDG-PET/CT is better in restaging setting than CT or MRI alone in characterization of equivocal CT masses, where mixed type of lesions pose difficulty in restaging as seen in one of our patients with an equivocal cystic lesion on CT alone (Figure 3). A non-significant FDG uptake was seen in the lesion excluding any possibility of malignancy. 

Our study had some limitations related to small numbers of patients and lack of statistical analysis. The primary objective of the study is to describe the spectrum of imaging findings in high risk testicular cancer patients in restaging settings Assessment of sensitivity, specificity and impact on patient management is beyond the scope of this study. Our own initial experiences suggest that the ^18^F-FDG PET-CT is useful in restaging and provide prognostic information in high risk testicular cancer patients. 

In conclusion, ^18^F-FDG PET/CT scan is a time-tested tool for evaluation of oncological disease management especially in follow up and restaging. Our results show that ^18^F-FDG PET CT scan effectively helps in characterization of difficult/equivocal lesions and helps in changing management decisions most often. In our study the decisions were also changed or augmented in (25%) of the cases where there were dual oncology opinions of wait and watch or go for treatment option. Restaging scans characterized the high value lesions and showed high SUV values suggesting tumor viability. ^18^F-FDG PET-CT scan in a restaging setting may be an excellent tool for follow up of disease however the restaging should be tailored on patient to patient basis to get maximum benefit of this high cost imaging modality which have limited availability but high efficacy.
